# Cryptococcoid Sweet syndrome: a case report

**DOI:** 10.3389/fmed.2024.1468712

**Published:** 2024-10-09

**Authors:** Martina Volonté, Giacomo Fiandrino, Camilla Vassallo, Stefania Barruscotti, Chiara Giorgini, Carlo Francesco Tomasini, Valeria Brazzelli

**Affiliations:** ^1^Institute of Dermatology, Fondazione IRCCS Policlinico San Matteo, Pavia, Italy; ^2^Pathology Unit, Fondazione IRCCS Policlinico San Matteo, Pavia, Italy; ^3^Department of Clinical, Surgical, Diagnostic and Pediatric Sciences, Institute of Dermatology, Università degli Studi di Pavia, Pavia, Italy

**Keywords:** acute febrile neutrophilic dermatosis, cryptococcoid acute febrile neutrophilic dermatosis, cryptococcoid Sweet syndrome, neutrophilic dermatosis, Sweet syndrome

## Abstract

Cryptococcoid Sweet syndrome (cSS) is a recently described clinical and histological variant of Sweet syndrome (SS). Its cutaneous presentation is similar to the classical form of SS but it includes atypical findings, such as capsular and yeast-like structures on microscopy that are reminiscent of *Cryptococcus* species. However, in cSS, fungal staining and cultural examination are negative, whereas myeloperoxidase (MPO) staining on biopsy specimens is typically positive. Due to the rarity and the diagnostic challenge represented by this disease, its extracutaneous involvement, and the latency in its diagnosis, this condition is frequently associated with poor prognosis. In this study, we report the case of a cSS patient with a positive outcome.

## 1 Introduction

Sweet syndrome (SS) is a reactive neutrophilic dermatosis of unknown origin, often associated with infections, medications, or malignancies (1–3). It typically presents as an acute eruption of tender erythematous-edematous plaques or papules. It is frequently accompanied by systemic symptoms and laboratory abnormalities, such as fever, constitutional symptoms, leukocytosis, and elevated inflammatory markers (2). SS has a broad differential diagnosis, as it can mimic other inflammatory diseases, cutaneous malignancies, or infections (2). Over time, various subtypes of SS have been identified from both clinical and histological perspectives (2).

Cryptococcoid Sweet syndrome (cSS) is a recently described clinical and histological variant of SS (1). Its cutaneous presentation is similar to the classical form of SS but includes atypical clinical findings, and capsular and yeast-like structures on microscopy that are reminiscent of *Cryptococcus* species (1). However, in cSS, fungal staining and cultural examination are negative, whereas myeloperoxidase (MPO) staining on biopsy specimens is typically positive (1).

Due to the rarity of this disease, the diagnostic challenge it presents, its extracutaneous involvement, and the delays in diagnosis, this condition is often associated with poor prognosis (1). Herein, we report the case of an antineutrophil cytoplasmic antibody (ANCA)-positive patient who rapidly developed cutaneous lesions and experienced a deterioration in general conditions. The patient was diagnosed with cSS and achieved a positive outcome.

## 2 Case report

A 57-year-old man was transferred to our center from a peripheral hospital due to a rapid worsening of preexisting multifactorial anemia (hemoglobin 5.7 g/dL) associated with melena of unknown origin, despite the extensive diagnostic workup performed (colonoscopy, gastroscopy, and capsule endoscopy). The patient had a history of pauci-immune p-ANCA-associated crescentic glomerulonephritis, leading to end-stage chronic kidney disease. He had been on peritoneal dialysis for 1 year prior to presentation. He had been treated with steroids without achieving ANCA negativization. His medical record also included emphysematous chronic obstructive pulmonary disease (COPD), hypertension, chronic gastritis, and depression. At the time of hospital admission, he was receiving therapy with prednisone 5 mg twice a week, escitalopram 10 mg/day, antihypertensive medications (bisoprolol 2.5 mg/day, ramipril 5 mg/day, and doxazosin 8 mg/day), and supplementation therapy with iron, folic acid 7.5 mg/day, and erythropoietin 10,000 IU subcutaneously twice a week.

**Figure 1:**
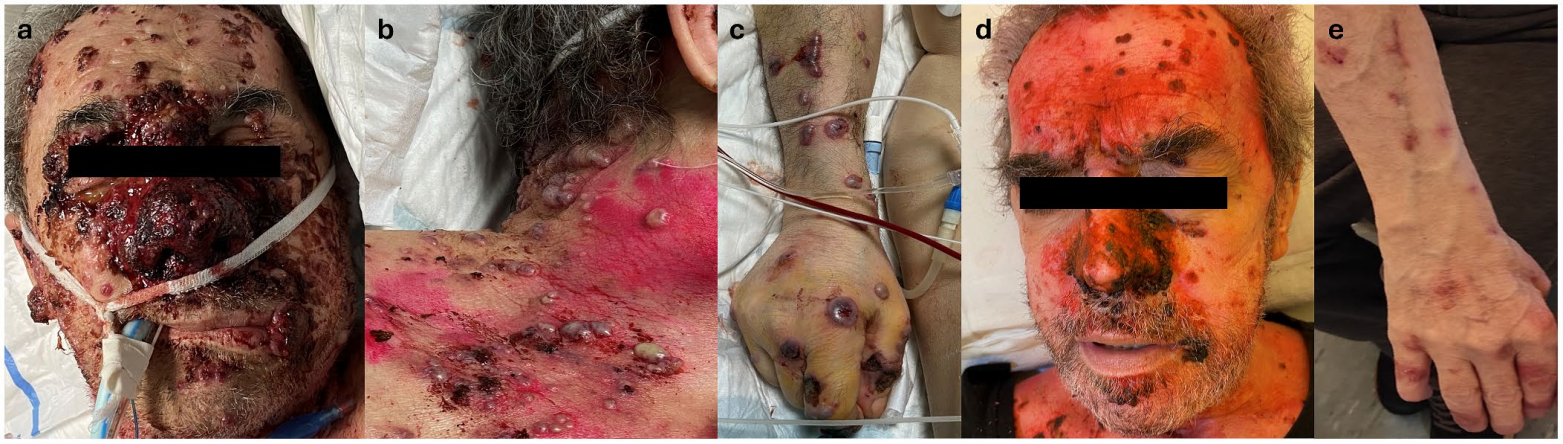
**(a)** Clinical appearance of the face, showing extensive necrotic tissue involving the middle and nasal area and discrete necrotic nodular lesions on the forehead and cheeks. Note nodular necrotic lesion on the lips. **(b)** Clinical appearance of lesions on the chest: edematous hemorrhagic papulo-pustules with tendance to punched-out ulceration. **(c)** Clinical appearance of lesions on the right arm: bullous hemorrhagic nodules showing umbilicated morphology with tendance to punched-out ulceration. **(d)** Clinical appearance of the face 10 days after introducing high-dosage systemic corticosteroids, with great improvement of lesions without scarring. **(e)** Clinical appearance of the right arm approximately 20 days after the introduction of high-dosage systemic corticosteroids, with almost complete remission without scarring.

During his hospital stay, pulmonary consolidation involving the right upper lobe, with pleural effusion and enlarged mediastinal lymph nodes, was detected, associated with an increase in inflammatory markers. Suspecting an infectious origin, systemic empirical antibiotic therapy (levofloxacin, later switched to piperacillin/tazobactam) was administered. A few days later, the patient developed disseminated hemorrhagic edematous cutaneous lesions with vesiculation and necrotic trend toward punched-out ulcerations, primarily on the head and, to a lesser extent, the trunk and limbs, where they appeared more scattered and with an umbilicated morphology. Two days after the onset of cutaneous manifestations, the patient was transferred to the intensive care unit (ICU) due to a deteriorating general condition, with respiratory failure and fever requiring invasive ventilation. The cutaneous manifestations worsened, with extensive skin necrosis, particularly in the nasal area, covered by granulation tissue and hemorrhagic and bullous lesions (Figures 1a–c). Erosive lesions involved the tongue, oral, and nasal mucosa with significant crusting of the lips. A biopsy was taken from the right arm for histological examination, cultures, and direct immunofluorescence (DIF). Systemic therapy with antifungal and antiviral agents was initiated, and antibiotic therapy was continued. However, the patient continued to deteriorate.

An extensive infection workup was performed, including blood cultures, cutaneous swabs, serology, and/or polymerase chain reaction (PCR) for human immunodeficiency virus (HIV), hepatitis, human herpes virus (HHV) 6, HHV 7, HHV 8, varicella-zoster virus (VZV), herpes simplex virus (HSV) 1, HSV 2, cytomegalovirus (CMV), Ebstein–Barr virus (EBV), leishmaniasis, toxoplasmosis, cysticercosis, and *Mycoplasma pneumoniae*. Cutaneous swabs for bacterial and fungal cultures were persistently negative, as was β-D-glucan. In contrast, respiratory cultures from bronchoalveolar lavage (BAL) grew *Candida albicans* (100 UFC/mL), which is considered a non-specific finding. Routine blood examinations showed neutrophilic leukocytosis [11.61 × 10^3^/μL, normal values (n.v.) 4.00–10.00 × 10^3^/μL; neutrophils 9.8 × 10^3^/ μL, n.v. 2.0–8.0 × 10^3^/μL] with increased reactive C-reactive protein (CRP) (17.41 mg/dL, n.v. <0.5 mg/dL) and other inflammatory markers. A comprehensive autoimmunity panel, including antinuclear antibody (ANA), extractable nuclear antigen (ENA), lupus anticoagulant (LAC), and complement levels, showed only positivity for p-ANCA and MPO (19.0 U/mL, n.v. <5.0 U/mL) and low C3 levels (57.0 mg/dL, n.v. 75.0–140.0 mg/dL).

**Figure 2:**
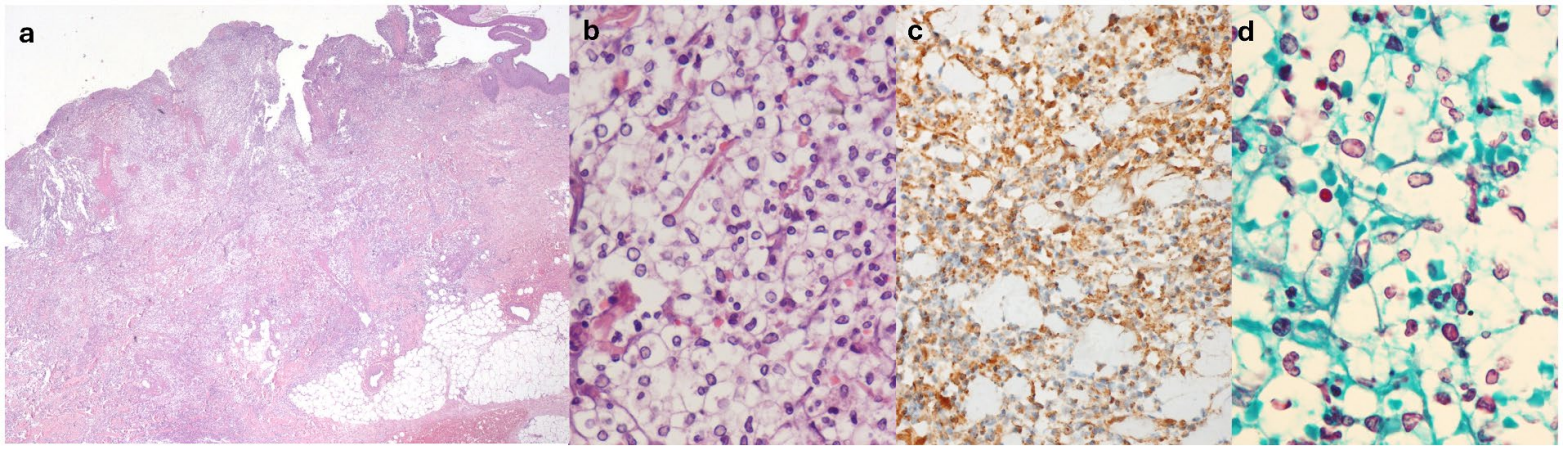
**(a)** Hematoxylin-eosin, original magnification 2.5×: diffuse dermal cellular infiltrate. **(b)** Hematoxylin-eosin, original magnification 60×: rounded basophilic bodies surrounded by clear vacuolated spaces, admixed within mature polymorphous nucleated neutrophils. **(c)** Myeloperoxidase (MPO) staining (positive), original magnification 40×. **(d)** Grocott methenamine silver (GMS) staining (negative), original magnification 60×.

Histological evaluation of the skin lesions revealed interstitial and perivascular cuffs of mononucleated elements with round nuclei and clear, abundant cytoplasm, resembling basophilic yeast-like structures surrounded by clear vacuolated spaces (Figures 2a,b) These elements expressed MPO (Figure 2c), cluster of differentiation 15 (CD15), and vimentin, and exhibited variable staining for CD45/LCA, CD14, and CD35. These findings primarily indicated elements of the granulocytic lineage. Abscess collections and macrophage accumulations (CD68/PGM1^+^, CD163^+^) were also present at the periphery of the infiltrates. Moreover, although vasculitic changes were evident within the infiltrate, vascular structures distant from it showed no significant inflammation or structural alterations. Stains, including Gram, Warthin–Starry, periodic acid-Schiff (PAS), Grocott’s methenamine silver (Figure 2d), and mucicarmine, as well as tissue cultures for fungal, bacterial, and mycobacterial microorganisms, were negative. DIF showed mild positivity for C3c and IgG in a perivascular distribution—these findings were considered insignificant by the pathologist. The clinical, morphological, and immunophenotypic characteristics were consistent with cSS.

**Figure 3:**
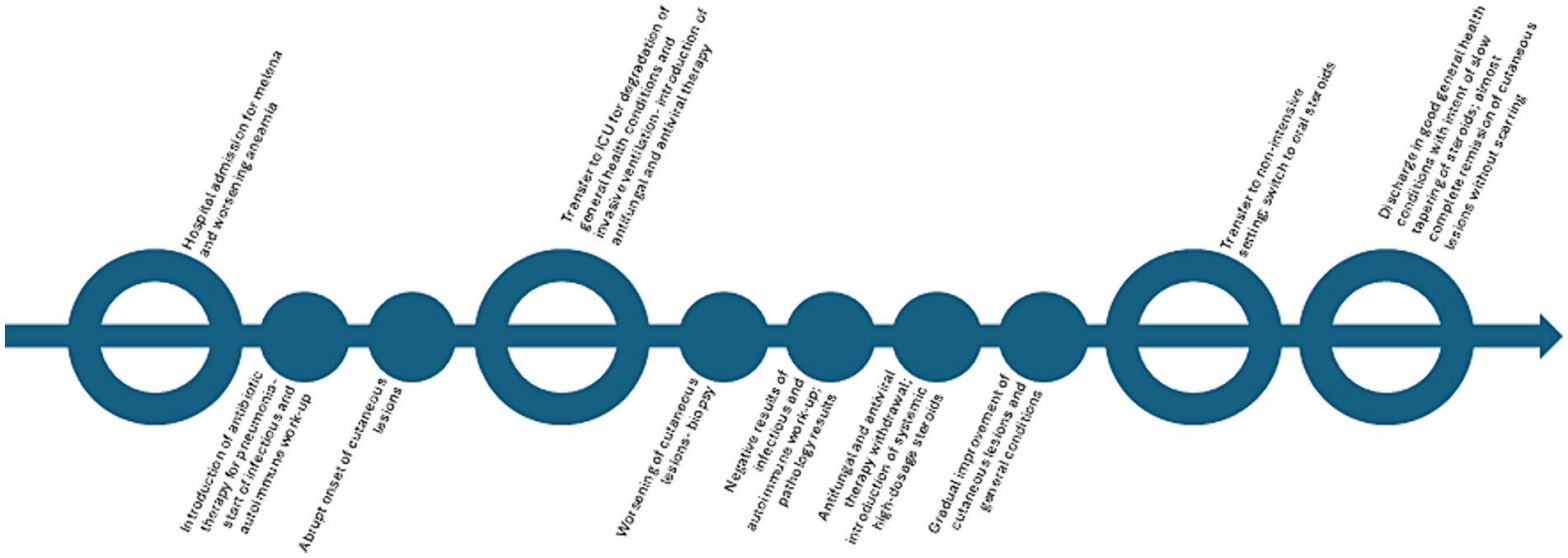
Timeline of the episode of care.

Antiviral and antifungal therapy was discontinued following biopsy results, and wide-spectrum systemic antibiotic therapy was maintained. After introducing high-dose systemic steroids (methylprednisolone 40 mg/daily intravenous), along with local antibiotics/steroidal cream (gentamicin and betamethasone cream 0.1% + 0.1%) and non-adherent dressings, the patient showed progressive improvement (Figures 1d,e), subsequently reaching complete remission of skin lesions without scarring in approximately a month. The patient was later discharged with only minimal adjustments to his previous home therapies, and he was switched to oral prednisone (25 mg/daily), which was slowly tapered to the previous chronic dosage of 5 mg/twice a week in 8 months without recurrences, with good compliance from the patient (Figure 3).

The patient reported a sense of wellbeing and satisfactory quality of life with the home therapy and continued to attend scheduled visits. He also reported that complete remission of the cutaneous lesions without sequelae seemed inconceivable, given the severe disfigurement during the acute phase. However, in the initial months, the patient reported being fearful of a possible recurrence from a dermatological and general health perspective.

At a 3-year follow-up, the patient had not experienced any recurrences of cSS, despite worsening general health. He died a few weeks after the last follow-up visit at the age of 61 years from septicemia unrelated to cSS.

## 3 Discussion

SS, or acute febrile neutrophilic dermatosis, is a reactive cutaneous condition associated with the acute onset of tender and edematous papules, nodules, and plaques (2–4). Three subsets of SS have been described: the classical form, the malignancy-associated form, and the drug-induced form (4). Additionally, various SS clinical and histological variants have been recognized over time (2).

The onset of SS usually follows a gastrointestinal or upper respiratory tract infection, pregnancy, inflammatory bowel disease, or autoimmune diseases (classical variant). However, it can also be associated with hematologic or visceral malignancy (malignancy-associated variant) and medications (drug-induced variant) (2, 4, 5).

Various diagnostic criteria for SS have been proposed. The first criteria were established in 1986 by Su and Liu (6) and reviewed by von den Driesch (7) in 1994. In this set of criteria, the diagnosis of SS relies on the fulfillment of two primary criteria (acute onset of tender erythematous cutaneous papules or plaques and the presence of a dermal neutrophilic infiltrate) and at least two of the four minor criteria (fever, leukocytosis, response to corticosteroids, and association with infection, malignancy, or other underlying conditions) (2, 6). Once incorporated into the original diagnostic criteria, the revision performed by Nofal et al. (8) in 2017 has excluded the absence of leukocytoclastic vasculitis as mandatory for the diagnosis of SS. It has sparked debate on its role as an epiphenomenon or a pathogenetic step (2, 9, 10). A previous study by Malone et al. (11) investigated the association between vasculitis and SS and highlighted that 29% of patients diagnosed with SS met the criteria for true vasculitis.

CSS is a rare entity first recognized in 2017 by Wilson et al. (2) in a case series reported by Ko et al. (12) 4 years earlier. It presents with clinical lesions resembling those of classical SS with some atypical features, such as umbilicated nodules, extensive mucosal involvement, and microscopic findings of pseudocapsulated- and yeast-like structures mimicking *Cryptococcus* species (5, 12).

To the best of our knowledge, only 17 cases of cSS have been reported in the literature (Supplementary Table S1), 11 of which were recognized in female patients with an average age of 70.5 years and one in an 18-year-old girl (1, 13). Of the 5 cases appearing in male patients (including the present one), the average age was 63.0 years (1).

From a clinical perspective, cSS presents with a sudden onset of erythematous-violaceous plaques and/or edematous umbilicated papules with a molluscoid appearance and nodules with a necrotic course (resembling cutaneous cryptococcosis), sometimes with bullous hemorrhagic aspects, involving the oral mucosa more frequently than its classical counterpart (1, 14). Moreover, limb and acral involvement has been described more commonly in the cryptococcoid variant than in the classical form (1, 4).

Among the most important differential diagnoses are infectious diseases (such as cutaneous cryptococcosis, fusariosis, disseminated herpes zoster, or secondary syphilis), cutaneous and systemic vasculitis, bullous lupus erythematosus, erythema multiforme, granulomatous diseases, cutaneous malignancies (such as lymphoma and leukemia cutis), and exogenous dermatoses, such as alkali burns and iododerma (2, 15). Notably, although rare, the latter may also present as cryptococcoid neutrophilic dermatosis from a histological point of view: in the present case, iododerma was excluded since no exposure to iodide was recognized in our patient before the onset of cutaneous lesions (15).

The diagnosis of cSS is based on histological findings and immunohistochemical stains in the setting of negative cultures/identification of the microorganism through PCR or antigenic tests and negative microorganism stains (1). From a histological point of view, cSS is characterized by a dermal inflammatory infiltrate with neutrophils, variable edema of the papillary dermis, vacuolated spaces, and basophilic acellular bodies. The latter two characteristics resemble the capsule and the budding yeast form of *Cryptococcus*, respectively (1, 12).

The most important clue to the diagnosis relies on histological examination with MPO stain, which appears to be positive in cSS. On the other hand, PAS and other stains, such as Grocott methenamine silver (GMS), mucicarmine, gram, and Fontana-Masson, are negative in cSS. When available, fluorescent *in situ* hybridization (FISH) with *Cryptococcus* specific-probe tests negative (16). When performed, DIF is usually reported as negative (1).

The pathogenic mechanism, both clinically and histologically, is not yet well-defined. However, following the hypotheses proposed by Sherban et al. (14), we believe that bullae formation with dermoepidermal splitting could result from the marked activation of neutrophils. This activation is likely related to the expression of mediators, such as CD3, CD163, tumor necrosis factor-alpha (TNF-α), interleukin (IL)-8, IL-17, matrix metalloproteinases (MMP)-9, and MPO, which may have a lytic role (14). From a histopathological perspective, the peculiar aspect of basophilic bodies surrounded by clear vacuolar spaces mimicking the capsule and the yeast form of *Cryptococcus* could be due to the process of neutrophil degradation, controlled by a mechanism of autophagy-related programmed necrotic death involving extensive cytosolic vacuolization (2, 12). Additionally, in the literature, there are some overlapping cases of cSS and the histiocytoid variant, as reported by Wilson et al. (5) in 2014 and Ko et al. (12) in the original series.

In our case, although vasculitis (particularly ANCA-associated forms such as microscopic polyangiitis and granulomatosis with polyangiitis) was considered in the differential diagnosis, the patient was ultimately classified as having cSS. We believed that the vasculitis-like changes, along with the doubtful perivascular positivity on DIF, represented an epiphenomenon within the context of cSS rather than a primary entity. The presence of vasculitis in SS is recognized as a consequence of noxious products released by neutrophils, especially in long-standing lesions due to prolonged exposure to harmful metabolites (11). Furthermore, despite the limited number of cSS cases reported in the literature, vasculitic changes were observed in other cases: focal vasculitis was found in the skin biopsy of the case reported by Stauder et al. (1); a dense perivascular neutrophilic infiltrate with prominent fibrinoid necrosis was noted in the case by Skaljic et al. (17); dense neutrophilic infiltrates with leukocytoclastic and fibrin deposition were described in the second patient in the case series by Sherban et al. (14). Notably, in the latter paper, it was suggested that the extensive destruction caused by neutrophils may be compounded in ANCA-positive patients (14). On the other hand, vasculitic changes in SS do not seem to be associated with immune-complex deposition, however, such deposits have occasionally been observed in other conditions traditionally considered unrelated to immune-complexes (e.g., granulomatosis with polyangiitis), suggesting instead that they might act as alternative triggers for vasculitic lesions (11, 18). This raises the possibility that the mild presence of C3c and IgG deposits in our case of cSS could play a similar role. Undoubtedly, further studies are needed to understand the relationship between vasculitis and cSS better, particularly since cryptococcoid elements have been reported in both SS and forms of cutaneous vasculitis, as noted by Fresco et al. (19).

Apart from the peculiar clinical and histological aspects, no specific diagnostic criteria have been proposed for the diagnosis of cSS (1). Regarding the present case, similarly to the other cases reported in the literature of cSS, our patient met the diagnostic criteria of SS (1, 8).

In the literature, a possible association has been recognized with using hydralazine before the onset of cutaneous and systemic manifestations of cSS in three cases (1, 5, 17). Additionally, three other reported cases involved patients who were cocaine users (2 active and 1 former user) (1, 2). However, data on domiciliary therapy were available in only a minority of cases described in the literature (6/17 cases) (1, 2, 5, 13, 17). Two cases with a possible association with recently introduced vancomycin have been reported by Byekova et al. (16) and Boyd et al. (20). Both cases showed improvement after the withdrawal of the suspected causative drug, along with the use of topical steroids; in one of the two cases, oral metronidazole was also administered. In our opinion, the improvement without systemic steroid therapy suggests a possible iatrogenic trigger in this case. Vancomycin was also reportedly administered in three other cases around the time of the cutaneous eruption (4, 5, 14). A case of cSS onset following therapy with piperacillin-tazobactam for diverticulitis was reported by Ko et al. (12) in the original case series. Additionally, two other patients received piperacillin-tazobactam around the time of cutaneous lesion development, both in combination with other antibiotic therapies, including vancomycin (5, 14). Regarding the present case, our patient was on a baseline steroid therapy at a low dosage (oral prednisone 5 mg twice a week) for a previously known p-ANCA associated glomerulonephritis; however, he developed cSS, concomitantly with an episode of severe worsening of multifactorial preexisting anemia in the setting of melena and pneumonia.

Notably, considering similarity to our patient, chronic kidney disease was identified in nine patients, of which five showed end-stage renal disease; additionally, chronic anemia was reported in the medical history of two cases (1, 2, 5, 13, 14, 16, 17, 20). Three patients suffered from pneumonia, similar to our case, and developed cutaneous lesions after or concomitantly with microbiological therapy; additionally, two other cases reported the presence of pulmonary infiltrates and respiratory distress (1, 5, 12, 14). Moreover, seven of the reported patients showed positive circulating p-ANCA, as in the present case, and the case reported by Mazzei et al. (13) recognized an “ANCA-associated vasculitis” without further specification in the patient’s medical history (1, 2, 14, 17). Respiratory failure during the acute dermatological stage was reported in another case by Byekova et al. (16), and the same patient also developed upper gastrointestinal bleeding before the onset of cutaneous lesions. Similarly, in our case, the patient showed respiratory failure, requiring intensive care 2 days after the lesions onset and melena. In our case, despite extensive diagnostic workup, the source of the bleeding could not be clearly identified. Given its rapid resolution without targeted therapeutic interventions, it was interpreted as a possible manifestation of the patient’s pre-existing chronic gastropathy. Regarding the pulmonary infiltrate accompanied by an increase in inflammatory markers and enlarged mediastinal lymph nodes, it was considered of infectious origin and treated with antibiotic therapy. No imaging findings suggested pulmonary involvement from vasculitis, despite the patient’s known ANCA positivity. A positivity for *C. albicans* on respiratory cultures was found in one of the two cases reported by Jordan et al. (4), as in our case, where it was considered an incidental finding.

The optimal treatment for cSS has not yet been established; however, it is believed to rely on systemic steroids, similar to classical SS, mainly prednisone or intravenous methylprednisolone (1, 21). In the literature, oral dapsone in association with systemic corticosteroids has also been reported in 5 cases, 4 of which showed improvement in the condition (1, 4, 14). The association of prednisone, mycophenolate mofetil, and dapsone has been used in 2 of 3 cases reported by Sherban et al. (14), resulting in improvement in one case and death of the patient in the other. A case of improvement of cutaneous lesions with the use of topical metronidazole has been reported in the original series by Ko et al. (12) in 2013; however, limited information was provided regarding the case.

Despite the extensive and destructive cutaneous and mucosal involvement in the acute phases, lesions tend to resolve without scarring in a few weeks to months. However, due to the severity of the disease, the difficulty in recognition, and the latency of diagnosis, it has frequently led to patient death in the cases reported in the literature. Therefore, greater awareness of this rare SS variant seems mandatory for clinicians and dermatopathologists to ensure prompt and optimal patient management.

## Data Availability

The original contributions presented in the study are included in the article/Supplementary material, further inquiries can be directed to the corresponding author.
