# Supplementary material for: Cryptococcoid Sweet syndrome: a case report
Source: Front Med (Lausanne). 2024 Oct 9;11:1468712. doi: 10.3389/fmed.2024.1468712 (PMC11496249; doi:10.3389/fmed.2024.1468712)
Supplement: Supplementary file 1 [file Table_1.docx]

**Supplementary Material: Table 1**

| **Case** | **Age/Gender** | **Anamnesis** | **Medications** | **Mucosal Involvement** | **Laboratory Findings** | **Histopathologic Findings (stains/DIF)** | **Treatment** | **Outcome** |
| --- | --- | --- | --- | --- | --- | --- | --- | --- |
| *Present case* | 57/M | Chronic anaemia, COPD, CKD, ESDR, hypertension, remote pneumothorax, admitted for worsening aneamia associated with melena, recent diagnosis of pneumonia after hospital admission | Prednisone 5 mg/twice a week, escitalopram 10 mg/die, antihypertensive therapy (bisoprolol 2,5 mg/die, ramipril 5mg/die and doxazosin 8 mg/die), and supplementation therapy with iron, folic acid 7,5 mg/die and erythropoietin 10.000 UI sc twice a week, recent use of systemic empiric antibiotic therapy for pneumonia (levofloxacine and piperacillin/tazobactam) | Nasal and oral mucosa (lips, tongue) | Negative cultures, serologies for microorganisms, beta-D-glucan, cutaneous swabs, autoimmunity except for positive p-ANCA, positive MPO, neutrophilic leukocytosis, elevated RCP, low C3, positive *C. albicans* on BAL | Positive MPO, CD15, vimentin, variable staining for CD45, CD14, and CD35, weakly positive DIF, negative tissue cultures, GMS, Gram, Warthin-Starry, PAS, and mucicarmine | Antifungal and antiviral therapy (discontinued after biopsy results), antibiotic therapy, high-dose systemic corticosteroids | Complete remission in a month without scarring. No recurrences |
| *Stauder et al (2024)*  ***(1)*** | 57/M | Acute renal failure on CKD, hypertension, DM, psoriasis, hypoxic respiratory failure former substance use disorder (cocaine, LSD and marijuana) | Amlodipin, aspirin, carvedilol, finasteride, glimepiride, hydralazine, insulin, losartan, metoprolol, tamsulosin | Oral mucosa (including lips) | Elevated inflammatory markers, positive ANA, anti-histone, anti-chromatin, cryoglobulins, p-ANCA, MPO, PR3, negative anti-Jo-1, anti-Scl-70, anti-Smith, anti-SSA, anti-SSB, infectious work-up for microorganisms | Positive MPO and lysozyme, negative PAS, GMS, AFB, mucicarmine and Gram stain, tissue cultures | Discontinuation of hydralazine, intravenous methylprednisolone and dapsone; oral prednisone tapered | Gradual resolution of lesions in 2 months without recurrences |
| *Jordan et al (2022)*  ***(4)*** | 81/F | DM, breast cancer | N/A  Recent antibiotic therapy with trimethoprim-sulfamethoxazole, and therapy with vancomycin and valacyclovir | Involvement of mucous membranes | Negative cryptococcal antigens, *Histoplasma* and *Blastomyces* antigens, blood and exudate cultures, HIV test, rapid plasma reagin, leukocytosis with neutrophilia, elevated creatinine, low calcium and albumin, malignant cells on pleural fluids, positive *C. albicans* on respiratory cultures | Positive MPO, negative PAS, GMS, mucicarmine, DIF | Intravenous hydrocortisone for refractory shock; antifungal, antiviral and supportive care | Transferred to hospice care for gram-negative septicemia |
|  | 87/F | DM, hypertension, CKD | N/A | Not reported | Leukocytosis with neutrophilia, elevated creatinine, negative blood and stool cultures | Yeast-like structures weakly positive on GMS, negative PAS, DIF, tissue cultures | Empiric vancomycin and cefepime (discontinued after biopsy results), high-dose prednisone and oral dapsone, topical clobetasol; oral prednisone tapered at discharge | Gradual resolution of lesions in 1 month |
| *Alshaikh et al (2022)*  ***(21)*** | 63/M | N/A  *(“no significant medical history”)* | N/A | Not reported | Negative cultures on blood, urine, cerebrospinal fluid, serum cryptococcal antigens | Positive MPO, CD15, negative PAS, Grocott stain, tissue cultures | Systemic steroids *(suggested as answer to “what is the most appropriate treatment for this patient?”)* | N/A |
| *Mazzei et al (2020)*  ***(13)*** | 18/F | Pyoderma gangrenosum and SS, ANCA associated vasculitis, malignant arterial hypertension, ESRD | Azathioprine, prednisone, losartan, TMX, valacyclovir | Not reported | Leukocytosis, negative antigenemia for *Cryptococcus* | Positive MPO, negative “special stains”, negative cultures | Systemic steroids (methylprednisolone) | Resolution of lesions |
| *Sherban et al (2020)*  ***(14)*** | 70/F | ESRD, DM, COPD, recent diagnosis of *Pseudomonas* pneumonitis and parotitis, complicated by septic shock and respiratory distress requiring intubation | N/A  Multiple recent antibiotics (vancomycin, cefepime, doxycycline, cephalexin, cefazolin, piperacillin/tazobactam) | Not reported | Positive p-ANCA, PR3 | Negative PAS, GMS, Gram stain, DIF | Dapsone and prednisone (slow tapering) | Eventual clearance of skin lesions |
|  | 68/F | Chronic anaemia, hypertension, hypercholesterolemia, admitted and intubated for retropharyngeal abscess and respiratory distress | N/A  Intravenous antibiotics during hospitalization | Not reported | Positive p-ANCA, MPO, equivocal PR3, negative c-ANCA | Negative PAS, GMS, acid-fast bacilli test, Gram stain, DIF, tissue cultures | Prednisone, mycophenolate mofetil and dapsone | Death from urosepsis and multiorgan failure |
|  | 70/F | ESDR, DM, recent cardiac catheterization, admitted for RSV pneumonia | N/A | Oral mucosa (tongue) | Positive MPO, ANA, anti-histone, negative PR3 | Negative Gram stain, tissue cultures for acid-fast bacilli and fungi | Prednisone, mycophenolate mofetil and dapsone | Improvement |
| *Skalijic et al (2019)*  ***(17)*** | 70/F | Hypertension, CKD, DM, remote bladder cancer with prior cystectomy and diversion complicated by ureteral strictures | Amlodipine, aspirin, clonidine, clopidogrel, furosemide, hydralazine, insulin, simvastatin | Oral mucosa, eyelid ectropion | Elevated ESR/RCP, positive ANA, anti-histone, anti-dsDNA, p-ANCA, MPO, leukocytosis, normocytic anaemia, increased creatinine, haematuria with no red blood cell casts, negative serology and cultures, negative bacterial culture and viral PCR on eye exudate, negative RF, anti CCP, cryoglobulins, anti SSA/SSB, anti-Smith | Positive MPO, CD68, negative PAS, mucicarmine, DIF, tissue cultures | Hydralazine withdrawal, initial therapy with oral prednisone 60 mg/daily, then shifted to intravenous steroids (solumedrol 500 mg/daily), shifted back to oral prednisone (started at 60 mg/daily) slowly tapered at discharge | Gradual resolution of lesions, death within weeks after discharging due to age and comorbidities |
| *Wilson et al (2017)*  ***(2)*** | 48/F | Anaemia, cocaine use | None | Oral mucosa | Leukopenia with mild lymphopenia, normocytic anaemia, elevated ESR/RCP, positive ANA, p-ANCA, anti-SSA, low C3/C4, negative anti-SSB, anti-RNP, anti-Smith, anti-dsDNA, anti-CCP, cryptococcal antigen on serum and cerebrospinal fluid, cultures for VZV and HSV, infectious work-up for HIV, HBV, HCV, syphilis, histoplasmosis and coccidiosis | Positive MPO, negative PAS, DIF, tissue cultures | Empiric therapy with amphotericin B, high dose oral prednisone (discharged with intent of slow tapering) | Initial improvement, lost to follow up |
|  | 55/F | Chronic HCV, rheumatoid arthritis, cocaine use | None | Oral mucosa | Leukopenia with mild neutropenia and lymphopenia, normocytic anaemia, elevated ESR/RCP, positive p-ANCA, low C4, normal C3, negative ANA, anti-CCP, cryoglobulins | Positive MPO, CD68, negative PAS, tissue cultures, | Oral prednisone (slowly tapered at discharge) | Resolution of lesions without recurrences |
| *Byekova et al (2014)*  ***(16)*** | 82/F | DM, CKD | N/A | Oral mucosa (tongue and soft palate) | Elevated serum free-light chains | Negative PAS, GMS, mucicarmine, anti-*Cryptococcus* antibodies and FISH | Systemic corticosteroids, fluconazole | Death during hospitalization from multiorgan failure |
| *Wilson et al (2014)*  ***(5)*** | 75/M | Hypertension, DM, dialysis-dependent ESRD, CAD, recent finding of pneumonia | Aspirin, atorvastatin, clonidine, hydralazine, insulin, isosorbide mononitrate, lansoprazole, metoprolol;recent administration of piperacillin/tazobactam, ciprofloxacin, and vancomycin, shifted to aztreonam and linezolid for pneumonia | Not reported | Leukocytosis, elevated ESR/RCP, positive sputum cultures for MRSA, positive ANA (speckeled), anti histone, negative anti-Smith, anti-RNP, and anti-double strand DNA antibodies; negative fungal cultures (rare mixed bacteria skin flora) | Positive MPO, CD68/PG-M1, negative mucicarmine, PAS | Intravenous corticosteroids (methylprednisolone 40 mg/die) | Gradual improvement |
| *Boyd et al (2014)*  ***(20)*** | 63/M | Hemodialysis-dependent ESRD, COPD, CAD, recurrent *C. difficile* colitis | Numerous medications, recent (24 h before) introduction of vancomycin for colitis | Not reported | Normal white blood cells count | Positive MPO, lysozyme, CD15, negative PAS, GMS, mucin stains, DIF | Topical corticosteroids (triamcinolone 0.1% cream), vancomycin withdrawal, oral metronidazole | Resolution of lesions |
| *Ko et al (2013)*  ***(12)*** | 84/F | SLE, diverticulitis, recent finding of pulmonary infiltrates and nodules | Piperacillin/tazobactam for diverticulitis and pulmonary condition+ others | Oral mucosa (lower lip) | Positive multiple autoantibodies (unspecified) | Negative PAS, GMS, Fontana-Masson, mucicarmine | Intravenous corticosteroids (methylprednisolone) | Death during hospitalization (initial resolution of lesions, then recurrence) |
|  | 76/F | AML in myelodysplastic syndrome, recent finding of bilateral pleural effusion with interstitial oedema | N/A | Not reported | Leukocytosis, low haemoglobin, thrombocytopenia, promyelocytes, positive ANA | Negative GMS, PAS, Gram, mucicarmine, Fontana-Masson, MPO, CD34, CD117 | Prophylactic itraconazole and acyclovir; intravenous corticosteroids (methylprednisolone); chemotherapy for AML | Death during hospitalization for haemorrhage and respiratory failure |
|  | 79/F | CLL, small lymphocytic lymphoma, intermittent angioedema/urticaria | N/A | Not reported | N/A | N/A | Topical metronidazole gel | Improvement of lesions |

Supplementary Material-Table 1: current case and literature review.

*Abbreviations*: AML: acute myeloid leukemia; ANA: anti-nuclear antibodies; CAD: coronary artery disease; CCP: cyclic citrullinated peptides; CKD: chronic kidney disease; CLL: chronic lymphocytic leukemia; COPD: chronic obstructive pulmonary disease; DNA: deoxyribonucleic acid; GMS: Grocott–Gömöri methenamine silver; ESR: erythrocyte sedimentation rate; ESRD: end-stage renal disease; F: female; HCV: hepatitis C virus infection; LSD: lysergic acid diethylamide; M: male; MPO: myeloperoxidase; MRSA: methicillin-resistant *Staphylococcus aureus*; N/A: not available; PAS: Periodic acid–Schiff; p-ANCA: p-antineutrophil cytoplasmic antibodies; PCR: polymerase chain reaction; PR: proteinase 3; RCP: reactive C protein; RF: rheumatoid factor; RNP: ribonucleoproteins; RSV: respiratory syncytial virus; SLE: systemic lupus erythematous; TMX: trimethoprim-sulfamethoxazole.
